# Why are there conflicts of interest? Investigation of teacher exchange within Arts Education Group in China

**DOI:** 10.1016/j.heliyon.2023.e18985

**Published:** 2023-08-07

**Authors:** Xue Xia, Yi-Ning Yang

**Affiliations:** Northeast Normal University, Faculty of Education, 5268, Renmin Street, Changchun City, Jilin Province, China

**Keywords:** Teacher communication, Collectivized school-running, Conflict of interest, Teacher collaboration

## Abstract

The introduction of the collectivized school management mode marks a turning point for teacher exchange, however, it also brings numerous challenges. Whether or not breakthroughs can be achieved in teacher exchange hinges on the outcome of stakeholder negotiations. This study employs a qualitative approach through case analysis. Based on interviews with 15 members of the Education Group, game theory was employed to investigate conflicts of interest between exchange teachers and original teachers during teacher communication. This study aimed to stimulate backbone teachers' motivation to transfer to branch schools while providing a broader space for free exchange. The conflicts of interest in teacher exchange within educational groups were found to be closely associated with various interest subjects, objects, and institutional arrangements in the teacher exchange system.

## 1 Introduction

The demand for balanced development of quality education from the public is increasingly strong, and collectivized school management is an important measure to promote such balance. This reform has been implemented in many cities across China in recent years, and it shares similarities with the “Teaching School Alliance” that is currently popular in the UK. In 2010, the U.K. Department for Education released a white paper titled “The Importance of Teaching and Learning,” which aimed to establish a self-improving system [[1]] of schools known as the Teaching School Alliance, founded on an inter-school support framework. (U.K. Department for Education, 2010) states that “With the provision of government funding and accountability for evaluation, the Teaching School Alliance is responsible for teacher training, supporting member schools, and developing specialist teachers [[2]].”

A high-caliber teaching staff is essential to ensure the quality of education in schools. The allocation and quality of teaching resources are crucial factors that determine the effectiveness of collective school management. The teacher exchange system is a direct means to reduce disparities between schools, but stakeholder conflicts can impede its implementation. Whether communication among teachers in elite schools can achieve a breakthrough depends on the outcome of stakeholder interactions.

Therefore, to promote the transfer of expertise from backbone teachers to branch schools and facilitate cross-level communication, collaboration, and learning among teachers, this study employs a qualitative research design that focuses on analyzing the conflict of interest between exchange teachers and original teachers during teacher exchanges. By integrating game theory with teacher communication within the context of collectivized school management, the application of game theory in educational system reform is enhanced. Moreover, it can offer valuable insights into innovative exchange systems for collectivized school management reforms in China and other similar settings.

## 2 Literature review

The mandatory institutional change of the teacher exchange system is inevitably accompanied by conflicts of interest among different stakeholders during its implementation. This is due to the fact that the system affects various groups of teachers and schools, leading to a series of conflicts that may hinder its effectiveness. Teachers, as the target group for policy implementation, have been the focal point of scholarly research on the benefits of teacher exchange. One key consideration is their role as stakeholders. Qin& Yang (2010) argue that exchange teachers' personal values may not align with social values, and that there exist comparative interests - including economic, political, and cultural interests - in teaching at schools with significant differences in standards. They further suggest that high-quality schools offer more opportunities for professional development to teachers, facilitating the expression of their values [[3]]. Besides, teachers in communication will choose from three behavioral options - proactive, aloof, or passive - based on the cost-benefit principle [[4]].

The second issue pertains to the challenge of adaptation faced by exchange teachers as a target group for institutional change. Hohner& Riveros (2017) suggested that rural school teachers in southwestern Ontario, Canada are susceptible to loneliness due to factors such as the geographical location of schools and transportation issues, limited career development opportunities, and challenges in integrating with rural communities [[5]]. Teacher exchange programs have the potential to either mitigate or exacerbate disparities in teacher distribution, depending on which teachers transfer or exit the profession and who replaces them [[6]]. As a result, such programs may impose an additional burden on educators, adversely affecting their job satisfaction and well-being while also posing uncertain risks to educational outcomes.

In addition to educators, the impediments inherent in implementing teacher exchange programs in high-quality schools have been extensively studied. Teacher exchange is also viewed as a Robin Hood approach of “taking from the rich and giving to the poor" [[7]]. Carver-Thomas (2017) suggests that when teachers exchange between schools, the impact on the original school is equivalent to that of teachers leaving the school entirely [[8]]. This can have consequences for students' learning outcomes as well as academic and financial costs for the school. Zhang (2012) noted that teachers' communication has three main impacts on the benefits of quality schools: firstly, it increases the management burden of the school; secondly, it disrupts the inherent stable structure of school teachers; and thirdly, its uncertain influence on teaching quality is prominent [[9]]. Therefore, while it may contribute to narrowing the overall gap in teacher quality across schools of varying levels, conflicting objectives and ineffective incentives during implementation can still result in adverse effects. Throughout recent literature on the benefits of teacher exchange programs, scholars have predominantly approached the topic from economic and sociological perspectives. These studies often discuss challenges such as teachers' role readjustment and navigating the complex economic landscape in order to effectively promote successful teacher exchange systems. With the rapid development of group schooling nationwide, it is crucial to conduct research on the benefits of teacher exchange within this context. Such research can promote inter-school exchange and cooperation, as well as contribute to the overall development of prestigious school groups. However, currently there is a dearth of literature on this topic. The implementation of the teacher exchange system in the group is now more closely aligned with teachers' interests, yet conflicts between subject interests persist. These conflicts have resulted in numerous practical problems and serve as inherent obstacles to teacher exchange, cooperation, and professional energy diffusion within the group. Therefore, this paper examines the exchange of teachers within a group from both theoretical and practical perspectives, aiming to strike a balance between public interests and teachers' personal interests in order to mitigate conflicts among stakeholders.

## 3 Methods

### 3.1 Contexts

In recent years, the fervor for “school choice” continues to intensify amidst controversy. Elite schools tend to monopolize high-quality educational resources and students, prompting the emergence of collectivized school management as a transitional system. Hangzhou pioneered collectivized school management in 2004, with major cities following suit and placing their hopes on education reform centered around inter-school cooperation to facilitate the sharing of high-quality resources. This case study focuses on Shenyang Arts Education Group in China, which began operating regional public schools as a group in 2011. Over the years, it has achieved a high level of balanced education development that ranks among the top in the province. To enhance teacher training and promote growth for less qualified schools within the group, a teacher exchange system was implemented in 2018. The Arts Education Group's successful experience in selecting an implementation path, restructuring the system, and integrating multiple cultures has significant reference value.

In terms of the selection and assignment of exchange teachers, the education group has implemented a “Two-way Employment System for Exchange Teachers.” At the end of each semester, the group will compile statistics on two aspects: first, each school district should submit a demand table for exchange teachers to the group according to their actual needs; secondly, the group will disseminate this information to all teachers. The second initiative involves a survey of teachers' intentions for the upcoming semester, which challenges the traditional unilateral selection method that is typically dominated by school administrators. Positions are first established based on actual needs, and then teachers apply for corresponding roles according to their individual circumstances, thereby transforming the one-way employment process into a two-way selection process between schools and educators. Teacher salaries are determined based on position value. In such a two-way election system, both the leadership of the branch school and the front-line teachers have equal participation in the implementation of the exchange system, avoiding unilateral domination by the head school's leadership."

### 3.2 Selection of branch schools

**Fig. 1:**
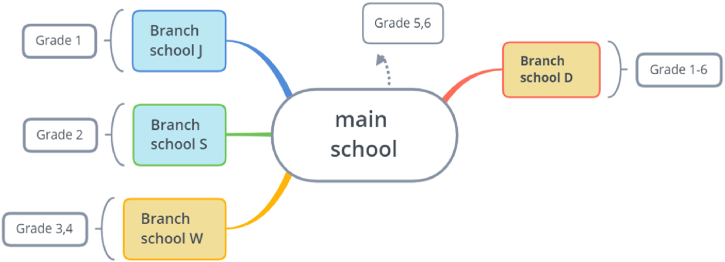
Location of main school and branch school.

By 2022, the group had had five campuses, with the D campus located more than 10 km away from the main campus in the suburban area of their city. The J, S and W branch schools are all situated within a 1-km radius of the main campus. Due to its association with the prestigious main school brand, enrollment at D school district had increased by over 2000 students in just seven years. The school is relatively large and encompasses grades 1 to 6, with the exception of Campus D. The three other branch schools are located within a 1-km radius of the main campus and have been integrated by grade level (as depicted in Fig. 1.).

### 3.3 Selection of paticipants

The teacher interviewees were categorized into three groups: four exchange teachers dispatched by the main school to the branch school (Z-D1, Z-D2, Z-W1, Z-W2), four exchange teachers dispatched by the branch school to the main school (J-Z1, J-Z2, S-Z1, S-Z2), and four non-exchange teachers dispatched by the branch school (D1, D2, W1, W2).

In order to mitigate the impact of grade-related factors, interviewees from D campus were selected randomly among teachers in grades 3 and 4 who align with W campus. Please refer to Table 1 the below for information on the selected participants.

**Table 1:** Basic information table of interviewees (teachers).

Interviewees	Teaching experience (years)	Age	Degree	Teaching subject	Head of Classroom	Backbone teacher
*Z-D1*	26	48	Bachelor	Math/Chinese	yes	yes
*Z-D2*	33	54	Bachelor	Math/Chinese	yes	yes
*Z-W1*	18	40	Bachelor	English	no	yes
*Z-W2*	14	36	Master	Math/Chinese	yes	yes
*J-Z1*	30	52	Bachelor	Science	no	yes
*J-Z2*	20	42	Bachelor	Math/Chinese	yes	yes
*S-Z1*	16	38	Bachelor	Integrated practice	no	no
*S-Z2*	21	43	Bachelor	Arts	no	no
*D1*	10	32	Bachelor	Math/Chinese	yes	no
*D2*	25	47	Bachelor	Math/Chinese	yes	no
*W1*	22	44	Bachelor	Math/Chinese	yes	no
*W2*	24	46	Bachelor	Science	no	yes

### 3.4 Data collection and analysis

This study employs a qualitative approach in the form of a case study as its research methodology, with semi-structured interviews serving as the primary means of data collection. The exchange of ideas among educators encompasses various subjects, and through the use of qualitative methods, this study aims to delve deeper into the psychological states and subjective motivations that underlie their construction of meaning. In the process of teacher exchange, both individuals and objects undergo continuous development and change over time. Through interviews, one can track the dynamic changes that occur over time and provide a detailed and comprehensive analysis of individual elements. The interviewees comprised of both principals and teachers, with the former providing insights on exchange teachers' work attitudes, practical roles, existing challenges within the teacher exchange system, as well as recommendations for improvement. Exchange teachers from the main school were primarily queried regarding their willingness to participate in an exchange program, interest requirements, adaptation upon entering branch schools, effects of various forms of exchange and communication, encountered difficulties during cooperation and exchange, as well as assessment and evaluation criteria. Before the interview, the researcher designed a broad outline of the interview. In the actual interview, the way of asking questions varied from person to person and situation to situation. Furthermore, the researcher maintained an impartial attitude, acknowledged diverse responses from interviewees, and probed further based on their perspectives. Due to the sensitive nature of this research, which involved conflicts of interest, a circuitous approach was adopted for conducting interviews that lasted between 40 and 90 min. With the permission of the interviewees, the original interview materials were preserved in the form of audio recordings. After the interview, the audio recording materials were transcribed into Chinese. Only quotes used in this current paper were translated into English. Next, the transcripts were read repeatedly many times for a complete understanding of the data. Thematic analysis was applied to analyze the qualitative interview data. The interview materials were stored, coded, indexed and analyzed by using the qualitative research software Nvivo 12 Plus.

## 4 Theory

### 4.1 A theoretical perspective: game theory

Game theory is a theoretical tool for analyzing the decision-making process of rational individuals in situations where mutual influence exists. The fundamental premise of game theory is that each player involved in a game acts instrumentally rational and always strives to maximize their own preferences within given constraints [10]. The benefits obtained by each participant are not solely determined by their individual choices but also depend on the decisions made by all other players, as they endeavor to optimize their own interests. Using game theory analysis enables the prediction of player behavior and equilibrium outcomes. This paper primarily focuses on non-cooperative games, assuming that each party involved in the game independently makes decisions based on their own interests, emphasizing individual rationality. We cannot get out of the prisoner's dilemma by denying individual rationality, but by improving the system to achieve the balance between individual and collective interests, so as to promote cooperation.

**Fig. 2:**
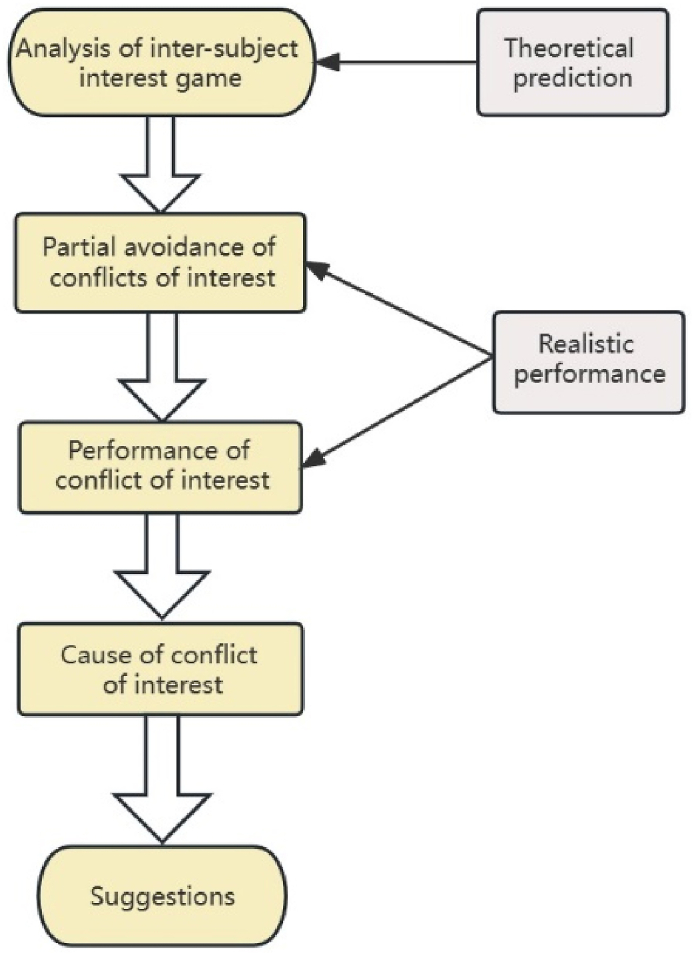
Theoretical framework.

Game theory, as the primary theoretical foundation of this study, is predominantly employed to examine how participants strategically select actions that optimize their own interests while being influenced by one another and taking into account policy information and relevant systems under realistic conditions. The theoretical framework is illustrated in Fig. 2.

Game theory can offer a robust theoretical framework for exploring the decision-making process and predicting the outcome of teacher exchange games among prestigious schools. Therefore, this study will employ game theory to theoretically investigate the intricate conflicts of interests faced by various stakeholders in institutional change processes.

#### 4.1.1 Interest conflicts behind teacher emotion

Teachers, being rational individuals, also adhere to the cost-benefit principle in their decision-making process and will opt for strategies or actions that maximize their benefits. They assess the costs and benefits before deciding whether to deeply cooperate with other teachers, only doing so when the expected benefits outweigh the expected costs. For individual educators, the benefits may manifest as admiration from peers, team accolades, or personal fulfillment. However, the high cost of communication can result in emotional conflict.

#### 4.1.2 The hidden “status” conflict behind teachers' identity consciousness

The various tiers of educational institutions possess distinct human capital, information, environment, and other resources that imprint the schools' signature characteristics deeply in society's collective consciousness. Teachers' identity conflicts during exchanges often conceal “invisible” status interests [10] such as self-esteem and prestige. Collaborative members employ various justifications to disguise them as conflicts related to tasks, procedures, and relationships. These include discrepancies in comprehension and perception of task content, purpose, procedures, and personality traits [11].

#### 4.1.3 Conflict of spiritual interests in the cultural conflict of teacher groups

When schools transition from independent to group-run organizational structures, the teacher culture may suffer due to conflicting strong and weak cultural influences, as well as the blending of external and endogenous cultures. Teacher culture refers to the system of values, ways of thinking, and behavioral norms that are meaningful to the teacher community within a school. This system reflects a consensus among teachers and is internalized in their teaching behaviors, work attitudes, and interpersonal interactions. It is also transmitted to all members of the school. Teacher culture develops over time and becomes deeply ingrained in teachers' behavioral styles and image identification systems.

Culture exhibits a certain degree of stability, and when external culture impacts teacher communication, the exclusivity of traditional teacher culture is subsequently heightened. The crux of cultural conflict among teachers lies in conflicting values, which are primarily manifested through differences in educational philosophy and working attitudes between different groups of teachers, as well as evident disparities in interpersonal patterns among them.

### 4.2 Partial conflict-of-interest avoidance

In the face of governance challenges such as clear differentiation between veteran and novice teachers, as well as cultural diversity, the group endeavors to facilitate exchange teachers' adaptation to different schools through measures including “befriending veteran and novice teachers,” “mentoring and partnering,” “project teams,” and “distinctive evaluation during transition periods.” The Education Group adopts “inclusiveness, collaboration and responsibility” as its guiding principles to transform teachers on each campus. Exchange teachers are integrated into various research groups responsible for different courses, with the aim of gradually instilling in them a sense of identification with the school's culture of “responsibility”, thereby stimulating their sense of purpose and mission.

## 5 Results

Teachers' communication within the group primarily takes the form of voluntary participation, supplemented by administrative assignments. The former refers to teachers engaging in exchanges among schools based on actual needs within the group. The latter involves the district Education Bureau implementing a set number of exchange teachers for the education group, which are then assigned according to internal plans. According to regulations set forth by the education bureau, high-quality schools are required to exchange no less than 10% of their teachers each academic year, with backbone teachers accounting for at least 20% of the total number of exchange teachers. Furthermore, this exchange program must last a minimum of two years. In order to standardize and streamline the teacher exchange process, Art Education Group has developed an “Exchange System for Teachers in Branch Campuses” that integrates teacher performance, selection, and professional title evaluation into the exchange process.

After multiple rounds of consultation and high-level design, the management team effectively mitigated and resolved some conflicts of interest in teacher exchanges by adjusting the internal governance system. However, these adjustments were insufficient to fully address all conflicts. Our investigation revealed that there are significant conflicts of interest among teachers in terms of their emotions, “status,” and culture during the process of teacher exchange.

### 5.1 Interest conflicts behind teacher emotion

The level and quality of education between the main school and branch school exhibit a significant disparity. This gap generates an invisible organizational boundary that fosters heterogeneity and exclusivity among schools, while simultaneously creating differences in behavior standards and work values among teachers. During the interview, teacher W1 of the branch school said

“Branch school teachers may feel envious and express disagreement towards their counterparts at the main school who provide students with ample and high-quality practice. Such reactions are common in professional settings."

Teachers from the main school implement their work in accordance with the original fast-working mode upon entering branch schools, which exerts “pressure” on teachers in these schools. This pressure stems from comparisons of teaching outcomes, additional efforts to adjust working conditions, and public opinions from parents, all of which prompt branch school teachers to either assimilate or resist them.

Exclusive emotions are also present in faculty workshops. Teacher Z-D2 noted that ***“There is always someone absent from every seminar … so it can be difficult to truly come together.”*** The internal heterogeneity of resources and working atmosphere among different schools precisely shapes the evident individual differences in work attitudes, cognition of cooperation, and vision. Therefore, it seldom addresses the fundamental aspects of education and teaching in inter-school collaboration, and falls short of achieving a seamless integration between core concepts and pedagogical skills. Teacher Z-D2 also noted during the workshop that “***We typically only discuss knowledge points; can I provide guidance on how to teach the class? They rarely inquire about teaching methods. Their standard is simply to avoid mistakes in class."***

The cost of exchange is evident. During the process of exchanging and collaborating with teachers at branch campuses, instructors at the main campus must allocate time and effort to mentor other educators amidst their demanding schedules, without being obligated to share their own valuable experience acquired through years of practice for minimal gain.

### 5.2 Recessive “status” conflicts behind teacher identity consciousness

One of the key objectives of teacher exchange programs is to enhance professional quality through resource and experience sharing, as well as fostering intellectual collisions. The Arts Education Group has established studios for renowned teachers across various disciplines, conducted team evaluations, and facilitated interactive communication in the form of mentoring to achieve its goal of providing professional guidance to branch school teachers. As the teacher S-Z1, who was transferred to the main school, remarked, ***“Your institution exudes an air of elitism while I am in charge of a regular school.”*** The status is self-sustaining and has a continuous impact that accumulates over time. However, due to differences in identity consciousness, behavioral characteristics, and psychological cognition among teachers from different schools during team workshops, it is challenging to eliminate gaps between the main school and branch schools. The exchange teacher J-Z2 from the branch school J exhibited a sense of helplessness and compromise regarding her identity as a teacher at a branch school during the interview.

“When I first arrived here, I presented some of my own summarized experiences and ideas. However, the teachers in the main school may have perceived me as an outsider and were hesitant to fully embrace my proposals. There still remains a sense of superiority and authority among them during discussions, with the head teachers typically taking charge. As such, I now spend most of my time listening attentively while occasionally posing questions to gauge everyone's thoughts.”

Different qualified schools possess varying resources, such as human capital, information, and school environment. Consequently, the symbolic features of a school have become deeply ingrained in society's collective consciousness [12]. These symbolic features create a sense of boundary that can lead to conflicts in teachers' identity awareness. This is reflected not only in the material environment and rules and regulations but also in their educational ideas and teaching methods. There are typically latent recessive “status” interests, such as self-esteem and prestige, involved in teacher exchanges. These interests are often concealed through various conflicts related to tasks, procedures, and relationships that teachers manage in reasonable ways. Conflicts often arise due to inconsistencies in understanding and perception regarding task arrangements, objectives, processes, and personality traits. These latent “status” interests are accumulated resources that require self-maintenance and have a sustaining impact [13].

In general, main school teachers hold a dominant position in teams and tend to exhibit a sense of superiority and authority over branch school teachers due to the high quality of their main school. Branch school teachers are typically placed in a passive and subordinate role, which subtly influences others' perceptions of their status through factors such as job titles, appellations, and speaking opportunities. The accompanying conflicts arising from recessive “status” will impede the in-depth cooperation among teachers within the education group. Consequently, the objective and increasingly strengthened boundary differences between qualified schools and ordinary (less qualified) schools, as well as the hidden “status” conflicts behind them, estrange inter-school relations and hinder conscious interaction and cooperation during teacher exchange.

### 5.3 Cultural conflicts among teacher groups

Each school has its own unique group culture, which deeply imprinted on her teachers. Such imperceptible ideas will also be brought into new organizations or groups along with teacher exchange. If the value concept of exchange teachers cannot be well integrated with the new environment, it will go against the organizational concept and cause contradictions and counteractive effects.

Under the influence of the culture of “responsibility”, most teachers in primary schools are highly motivated and actively engaged in their daily work. As a result, their work is often conducted at a fast pace, leading many to develop a strong sense of competition.

As teacher J-Z2 exchange from branch school J to the main school said, ***“The teachers at the main school are super active in joining all sorts of activities. The competition is pretty intense and everyone's really eager to show off their skills. In my old school, we weren't as proactive and usually got assigned to participate by the school leaders.”***

For branch teachers accustomed to a slower pace of work, cultural conflicts may arise when transitioning into a high-pressure working environment. Some branch school teachers may struggle with adapting to the faster pace of the main school.

“The good thing about the previous school (branch school) was that it felt like home. There is too much pressure to relax here. The students require the quality of the teachers to be very high. There are also a lot of activities, which require you to keep pace. I didn’t get used to it when I got here.”

The strong contrast of teacher group culture brings great psychological pressure to exchange teachers while they join the new schools. In the interview, some teachers from branch schools mentioned that

“Branch school teachers go home after work. In our main school, we often work overtime without regulations, and the competition among colleagues is fierce, so we have no time to chat.”

Culture, once established, possesses inherent stability. During teacher exchange programs, external cultures may impact traditional teacher culture; however, this only serves to reinforce the exclusivity of said culture. The crux of conflicts within teacher culture lies in conflicting values - specifically, differences in educational and work attitudes as well as interpersonal relationship patterns among different groups of teachers. In the process of cultural collision, teachers will think and behave based on their respective cultural positions and life situations. Cultural exclusion may occur if effective communication is lacking among teachers. Informal groups such as “cliques” may emerge as a manifestation of cultural exclusion, posing significant challenges to both the adaptability of teachers in the new environment and school board management.

## 6 Discussion

**Fig. 3:**
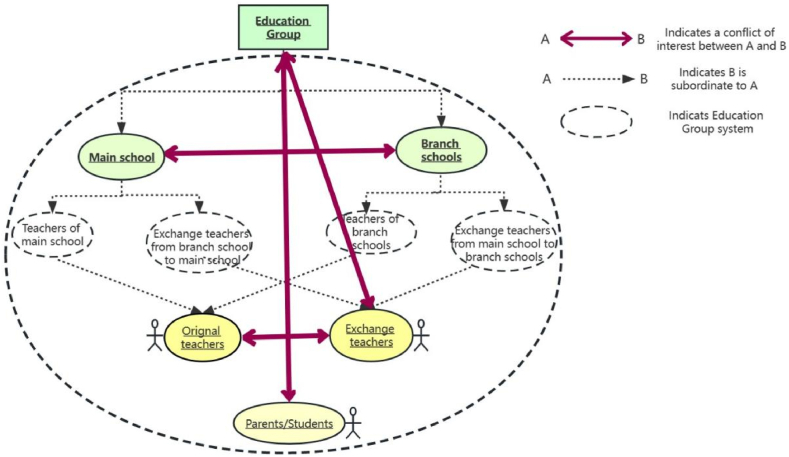
Interest relationship.

In light of the aforementioned specific manifestations of conflicts of interest, this section primarily delves into the underlying causes of such conflicts in teacher exchanges within the Arts Education Group, taking into account various stakeholders, objects of interest and institutional arrangements. The stakeholder diagram is shown in Fig. 3.

### 6.1 Stakeholders aspect: inconsistency of subjective motivation

Stakeholders have varying criteria for evaluating the benefits and costs of institutional change, based on differences in satisfaction levels and constraint conditions [ [14]]. During the implementation of a teacher exchange system, stakeholders engage in non-cooperative game relationships. This means that education group members are more likely to prioritize personal interests according to the “economic man” principle of action. Therefore, the public interests of educational groups are often overlooked.

#### 6.1.1 Inconsistency of value goals between main school and branch schools

As the executor of the teacher exchange system, the main school's value goal is to comply with and execute orders from superior administrative departments, enhance its educational standards, and expand and elevate its renowned brand. The precondition for the main school to actively assume a role in aiding and guiding teacher exchanges is that interest adjustments do not diminish its strength or core competitiveness. According to the interview results, Nvivo's coding node reveals that the primary codes related to the main school's interest appeal are: attrition of high-quality teachers, intricate school administration, elevated management pressure, exorbitant operational costs, teacher shortages, and student disparities.

The principal of the main school noted that teacher exchanges have resulted in a dilution of both teachers' and students' abilities, while the ever-expanding size of the school has also brought about significant management pressures. “***Because the whole group scale is too big, management pressure is very big now. It's so hard and exhausting to worry about losing control … The schools in charge of 9000 people are certainly not as careful as those in charge of 1000 people. I used to be able to name every teacher, get to know every teacher's strengths, have in-depth conversations with teachers, coach a lot of kids, talk to parents.*** That's not possible Now. ”

The principal of the main school is no longer able to provide meticulous humanistic care for teachers, offer specific guidance to students, or engage in deep conversations with parents to understand their true thoughts. Additionally, the value goal of branch schools is to acquire self-development and evolution momentum while bridging gaps between “resource demand” and “resource stock” in reality, thereby promoting teacher quality improvement. Based on the interview results, branch schools have two primary areas of interest: firstly, to recruit high-quality teachers who can effectively lead the development of less experienced colleagues; secondly, to ensure stability within their teaching faculty. The Arts Education Group allows exchange teachers to return to their original schools for at least a two-year period, demonstrating ethical respect for individual preferences. However, it also poses a significant challenge to the faculty stability of branch schools and their intricate teacher sourcing. The instability of the teaching team can impede the formation and development of a cohesive teacher culture, as well as hinder the cultivation of a sense of belonging and identity within the organization. From a student perspective, frequent teacher turnover can disrupt systematic implementation of lesson plans and prevent students from receiving comprehensive instruction. Meanwhile students have to adapt to new teachers passively. ***“Group exchange teachers can go back after two years, and many things have not been systematically implemented by teachers. Each teacher has his own way of playing, and the stability of the teacher team is a key factor for the development of a school.” (The principal D)***

#### 6.1.2 Inconsistency of interest between exchange teachers and received-school teachers

The inconsistency of interests between exchange teachers and host-school teachers is primarily manifested in the incongruity of interests between teachers from the main campus and those from branch campuses. Teacher exchange is a process of internalizing external social benefits for branch schools, where instrumental rationality plays a dominant role in stimulating continuous teacher exchange driven by self-interest. However, if branch schools are viewed solely as resource recipients, their unique resources may not be fully respected and effectively developed, thereby limiting the full potential of the “resource sharing” system performance (Wen, 2009)[ [15]]. In the process of improving less qualified schools (branch schools), the participation and energy levels of their members directly impact the effectiveness of quality improvement and connotation development. It is difficult to achieve a fundamental change in the quality of these schools relying solely on external forces. Attaching excessive importance to the value of the main school as an external entity, while disregarding the role and function of branch schools' insiders, will inevitably result in their inability to exchange ideas or cooperate with teachers from both institutions.

Secondly, some teachers from branch schools exhibit non-cooperative behavior in response to organizational change, primarily through passive participation and negative reactions. The benefits may include admiration, team recognition or self-fulfillment within the teacher group on an individual level. Additionally, their exchange costs are evident. Teachers from the main school are required to invest time and effort in guiding their peers through exchange and collaboration, even after a long day of work.

Considering individual rationality and interests, some exchange teachers view their exchange activities as “additional tasks that consume teaching time”, resulting in exchange benefits being outweighed by costs. Therefore, teachers tend to choose the optimal strategic action that is “most beneficial” to themselves, disregarding the collective rationality of overall education group development. This results in inconsistent efforts from both sides of the school in terms of exchange and cooperation.

#### 6.1.3 Inconsistency of interest between exchange teachers and education group

The participation of high-quality teachers in teacher exchange is a process of socializing teachers' self-value realization, in which value rationality plays a leading role. In the game process, as “economic men,” teachers tend to choose strategies that maximize their own benefits after weighing future expected benefits during institutional change. When the expected benefits exceed the expected costs, teachers are more willing to become active participants in the system. On the contrary, teachers will choose the behavioral strategy with lower risk and cost, which makes the game of all parties fall into the “prisoner's dilemma” during the implementation of the teacher exchange system, resulting in the inconsistency of interests between exchange teachers and education group.

Firstly, the distance cost arranged between school and residence is an important factor affecting teacher exchange. In the Arts Education Group, branch school D is relatively far from the main school. ***“It's too far. No one wants to come.“(Z-D1)*** Distance subsequently increases the cost of time-spending and inconveniences especially for teachers who need to care for children.

“I have kids to take care of, so I don’t want to go too far from my home.“(Z-W1) “It is not convenient to take care of the children. In the past, I could drop him off in the morning and take him to review what he has learned. Then it is easier to pick him up in the evening.” (S-Z1)

Secondly, the harmonious interpersonal relationship is directly related to whether exchange teachers can smoothly integrate into the new working atmosphere, which will affect their satisfaction degree of psychological and social needs. Teacher Z-D2 had concerns about relationships before her exchange, ***“What kind of people will I meet when I move to a new place? Will this kind of interpersonal relationship or new environment ostracize me? After all, I am in the minority. My disposition is understood in this familiar environment, and then I go to other schools, who can spoil me?”***

Finally, the need for professional development and occupation values is also one of the concerns for exchange teachers. Compared with the main school, each branch school can provide teachers with limited platforms and opportunities. Therefore, there is a big gap in the professional development and improvement space of branch schools for exchange teachers from the main school. As one teacher from the main school said, ***“There are more opportunities in the main school, such as student performances, and more opportunities for me to show, plus more opportunities to score points.” (Z-D1)*** In addition, there are apparent student differences among schools because of the gaps in students’ cognitive ability, socioeconomic status, academic performance, and so on. Compared with the main school, the self-realization of the professional value of branch school teachers is not easy to satisfy.

“It’s hard to get high marks for tutoring here.” (Z-W2), “Most students could not understand after once teaching no matter how hard I endeavor to teach. There’s no other way. I just do it to the best of my ability.“ (Z-D1)

#### 6.1.4 Inconsistency of interest between parents and education group

Changes in any educational system ultimately impact students and their parents. The presence of high-quality educators serves as a strong guarantee for the delivery of superior school education and teaching quality. As such, it is no surprise that nearly all parents aspire to enroll their children in top-tier schools where they can benefit from exceptional instruction and thrive within a supportive academic environment.

In the meantime, the educational group's public interest lies in achieving rational and equal allocation of educational resources for all members. Therefore, there exist divergent interests between individuals and the education collective. As direct advocates for their children's interests, some parents may object to teachers from either the main or branch school instructing their child's class. Such conflicts of interest between individuals and educational groups as a whole form resistance to the teacher exchange system.

### 6.2 Interest object level: scarcity of high-quality teachers

The scarcity of educational resources is the fundamental cause of interest conflicts. While the inter-school cooperation may bring a new turning point to branch schools under teacher exchange systems, it may also further dilute high-quality education resources in conditions where quality education resources are lacking, resulting in “lose-lose” rather than “win-win” outcomes. Scarcity “refers to the relationship between desire and its feasibility [16]. There are two primary factors contributing to the emergence of educational gaming due to resource scarcity: one is the constraint of educational resources, and the other is the insatiable demand from stakeholders [ [17]].

In the education group, high-quality teachers are a scarce resource, most of whom belong to the main school. When it comes to internal allocation of educational resources, all schools hope to acquire more qualified teachers in order to enhance their own popularity and competitiveness while supporting the operation of the education group. In practice, as the Arts Education Group continues to expand and parents become increasingly obsessed with prestigious schools, the influx of students has exacerbated the already prominent imbalance between high-quality teacher supply and demand.

Secondly, the demand for high-quality teachers from parents is constantly increasing. Parents of branch schools prefer their children to be taught by renowned educators from the main school with the expectation of receiving superior education. As stated by an exchange teacher in the interview, ***“At every parent-teacher meeting, parents secretly analyze whether I want to go back to main school.” (Z-D2).*** Simultaneously, there exists a reluctance among students and their parents to release their educators. Ultimately, the dearth of high-caliber instructors is what drives competition for educational resources between schools and families.

### 6.3 Institution level: defects of institutional arrangements

The conflict of interest arises from the inherent contradiction between individuals' interests and the mechanisms for realizing those interests, thereby highlighting a systemic flaw [ [18]]. It is an inherent defect in institutional approaches to realizing individual interests that intensifies conflicts among all stakeholders. The institutional arrangements for teacher exchange in the Arts Education Group are deficient in several aspects, including:

#### 6.3.1 Insufficient external system supply

The conflict of interest among stakeholders in the group can be attributed to the incongruity between system supply insufficiency and system selection objectives. Institutional supply refers to the capacity for creating, maintaining, and advancing a system towards its goals, which is limited by supplier's subjective preferences, interests structure, rationality level, and institutional environment [ [19]]. Under the circumstance of imbalanced distribution of high-quality educational resources, the objective of collective school management and teacher exchange system selection is to share premium resources among different schools and achieve mutual benefits. The involvement of educational bureaus has an impact on the positivity of school collaboration, which in turn affects the social repercussions of collectivization. In this game, the Education Bureau mandates that the leadership board of Arts Education Group assume responsibility for promoting educational resource balance. However, without an external supportive complementary system in place, interest conflicts within the education group are likely to intensify.

The reform of education investment mechanism is not yet fully implemented. Education bureaus have not established dedicated funds for the collectivization of education groups or teacher exchange, let alone a long-term investment mechanism. Given the realistic constraints on government funding, Arts Education Group primarily relies on its existing educational resources from the main school to support group operations. The implementation of teacher exchange requires a significant investment in costs, including school bus transportation, teacher subsidies, and training. However, due to the continuous expansion of the education group, internal allocation of exchange teachers within the group cannot meet the severe shortage of teachers. Therefore, the management board must independently secure funds to engage external personnel. However, due to relatively low remuneration, there is a high turnover rate among external teachers. One of the objectives of the teacher exchange system is to enhance teaching quality in less qualified schools. For branch schools, implementing this system may jeopardize group-wide teacher team stability given the aforementioned high turnover rate. Even the main school cannot guarantee an endless supply of qualified teachers. To ensure teacher stability, the main school must regulate the number of exchange teachers and limit high-quality teacher selection.

The reform of the teacher personnel system is lagging behind. As previously mentioned, the main school faces a dual challenge: on one hand, an abrupt increase in student enrollment has resulted in a shortage of teachers; on the other hand, regulations require that exchange teachers be dispatched to branch schools. However, education bureaus have not increased full-time teaching positions within educational groups. Consequently, educational groups must independently raise funds to hire teachers at low salaries. This also results in a decrease in the number of exchange teachers being dispatched to branch schools, which directly impacts the systematic development of teacher exchanges.

The third issue lies in the inadequate incentive and restraint mechanism. As pointed out by the principal of the main school, ***“There is no assessment, evaluation, reward or punishment for teacher exchanges within the group by the Education Bureau.”*** Although there are regulations on teacher exchange from educational bureaus, they have not been effectively implemented. So far, scientific indicators have not been used to evaluate the results of teacher exchange within the group. However, there has been a lack of corresponding incentive and restraint systems to motivate relevant parties to implement the teacher exchange system. Educational bureaus merely provide regulations and distribution schemes for resource allocation participants, which only fulfill the initial stage responsibility of the teacher exchange game. The entity has failed to fulfill its responsibility of assessing the appropriateness and feasibility of adopting government-provided regulations and allocation schemes for various stakeholders based on their respective circumstances, while also neglecting to clarify the situation and determine the extent of participants' responsibilities. As a result, regional education equity can only be achieved through personal beliefs and educational convictions held by group leaders.

“Most of the time, we are working for the government. For example, 43% of the external teachers in campus D, about 80, invest at least 3 million yuan a year. Who will solve the problem? Who will solve the cost of 2 million yuan spent by the commuter car every year? The school is responsible for it. This is too much for the government to bear. But now that the government can’t solve it, the problem is devolved to schools. The group’s financial problems are solved with the wisdom of these principals.” (Executive principal of school D)

#### 6.3.2 Limited incentive system of the internal education group

The incentive effects of the Arts Education Group's relevant system are limited. Specifically, the group's incentive system for exchange teachers includes preferential treatment in evaluating excellent teachers and recognizing outstanding ones. Regulated by school policies, teachers who demonstrate good or outstanding performance during the exchange period will receive additional points in the quantitative assessment of job evaluation and employment based on point allocation criteria. Additionally, two bonus points will be awarded to teachers' performance appraisal scale each semester. However, according to several exchange teachers, the allocation of two bonus points is deemed insignificant, particularly for experienced backbone teachers. As a result, it fails to serve as an effective incentive for main school teachers to participate in exchanges with relatively less qualified branch schools.

“There is a bonus policy, but the bonus is too insignificant to attract me.” (Z-D1)

“It’s a negligible percentage. Some teachers may total nearly 200 points and then add 2 points for communication.” (Z-W1)

Currently, the lack of incentive effects in regulations makes it challenging to effectively encourage teacher participation in exchanges.

#### 6.3.3 Insufficient compensation mechanism in education group

Currently, the education group provides limited options for compensating exchange teachers. For instance, Campus D is situated over 10 km away from the main school. Despite the provision of a “Warm School Bus” by the education group to facilitate teacher exchanges at Campus D, long-distance commuting still poses significant challenges to these educators. The education group also offered subsidies for exchange teachers during its early days, but these meager allowances were insufficient in comparison to the time and energy expended by such teachers. The compensation offered by education groups falls far short of the extra cost of teachers traveling to work. When the discrepancy between the loss of various costs and expected benefits of teachers' participation in intra-group communication is significant, it can negatively impact their communication intention, leading to a potential conflict between individual teacher interests and those of the group organization.

## 7 Conclusions

When it comes to the rational man hypothesis of teachers, the profession is often associated with notions of “selfless dedication” and being “engineers of the human soul”. However, solely emphasizing dedication without considering potential conflicts of interest can hinder effective implementation and result in a passive and formalistic approach. Improved systems are necessary to promote teacher exchange and education equity, making communication and mutual cooperation a rational choice for individuals seeking progress.

What can we do to reduce those conflicts? Here are four improvement suggestions.

The government and the group should enhance the incentive mechanism for teacher exchange. If feasible, the Group can provide more humanistic care for exchange teachers, such as facilitating their living or transportation. To motivate teachers' enthusiasm for exchange, school leaders could organize evaluation activities and give recognition to outstanding contributions. The weak schools can also improve their teachers’ professional development if back-bone teachers from good schools are encouraged to exchange there with individual research plan or school teaching development project.

Secondly, the teacher exchange program places greater emphasis on the involvement of middle managers. The main school can regularly organize training activities for group middle managers and arrange for inter-school teaching directors to rotate without disrupting the school's teaching schedule, thereby facilitating a wider convergence and diffusion of professional energy. During the interview, the researcher discovered that many teachers at the main school experienced unexpected growth through communication with their counterparts at the branch school, resulting in professional development. Starting anew in a different environment and among new colleagues presents an opportunity to showcase one's positive mindset and work ethic. In addition, teachers from the branch school who transfer to the main school not only gain access to a higher development platform and more opportunities for learning and communication, but also experience significant professional growth. Furthermore, this change in working attitude can provide enlightening help for their daily lives and education of children.

Thirdly, the group must redefine the cultural concept of teacher teams. A team for teacher exchange and collaboration can be established based on various cooperation needs, such as subject projects, curriculum research and development, interests and hobbies, interdisciplinary studies, and student requirements. The group should enhance the propaganda and mobilization of teacher exchange, disseminate the experience and perception gained from such exchanges to all teachers through various channels including teacher experience sharing meetings, so as to foster a positive public opinion atmosphere conducive to promoting teacher exchange work in the surrounding community.

In addition, teacher collaboration teams should be established as necessary. A team for exchanging and collaborating among teachers can be formed based on various cooperation needs, such as subject projects, curriculum research and development, interests and hobbies, interdisciplinary studies, and student requirements. With a shared goal and vision driving them forward, the teacher team fosters an environment of equal and interactive communication rather than the traditional hierarchical relationship between leaders and followers. In such a context, exchange and incoming teachers can effectively establish a professional learning community where they learn from each other, provide mutual assistance, and enjoy relatively equal status and discourse power.

There are still numerous deficiencies that require improvement and breakthroughs in this research endeavor. Due to objective constraints, I was unable to procure an adequate number of exchange teacher samples. By incorporating additional variables such as gender and qualifications, as well as supplementing quantitative research with qualitative analysis, the study will become more comprehensive and scientifically rigorous. Through this case study, I have identified new issues and points of interest that warrant further investigation and analysis. For instance, “what is the appropriate scale boundary for controlling elite school groups?” and “a comparative examination of teacher exchange programs between China and Britain in the context of collectivized schooling policies.” I look forward to exploring these topics in greater depth as part of my future research endeavors.

## Ethical approval consent

All subjects gave their informed consent for inclusion before participating in the study. The study was approved by Faculty of Education, Northeast Normal University, Changchun, 130024, China.

## Funding details

The research received no financial support.

## Author contribution statement

Xue Xia: Conceived and designed the experiments; Performed the experiments; Analyzed and interpreted the data; Wrote the paper. Yi-Ning Yang: Conceived and designed the experiments; Performed the experiments; Analyzed and interpreted the data; Contributed reagents, materials, analysis tools or data; Wrote the paper.

## Data availability statement

Data included in article/supplementary material/referenced in article.

## Additional information

Supplementary content related to this article has been published online at [URL].

## Declaration of competing interest

The authors declare that they have no known competing financial interests or personal relationships that could have appeared to influence the work reported in this paper.
