# Supplementary material for: Why are there conflicts of interest? Investigation of teacher exchange within Arts Education Group in China
Source: Heliyon. 2023 Aug 7;9(8):e18985. doi: 10.1016/j.heliyon.2023.e18985 (PMC10440518; doi:10.1016/j.heliyon.2023.e18985)
Supplement: Multimedia component 1 [file mmc1.docx]

| **Gathering Codes from all participants** | **Formulation of Codes** | **Sub themes** | **Themes** |
| --- | --- | --- | --- |
| High pressure, fast-paced, Strong team work sprit, Strong sense of competition, vigorous (main school) | Working Environment | Teachers' spiritual interests | Culture Conflict |
| Poor motivation to participate in activities, Difficulty in carrying out work, Ask for leave often | Working Attitude |  |  |
| Pay attention to the all-round development of students | educational concept |  |  |

**Themes Formulation**
